# Tactile Acuity Charts: A Reliable Measure of Spatial Acuity

**DOI:** 10.1371/journal.pone.0087384

**Published:** 2014-02-04

**Authors:** Patrick Bruns, Carlos J. Camargo, Humberto Campanella, Jaume Esteve, Hubert R. Dinse, Brigitte Röder

**Affiliations:** 1 Biological Psychology and Neuropsychology, University of Hamburg, Hamburg, Germany; 2 Instituto de Microelectrónica de Barcelona, Centro Nacional de Microelectrónica (CSIC), Bellaterra, Spain; 3 Neural Plasticity Lab, Institute for Neuroinformatics, Ruhr-University Bochum, Bochum, Germany; 4 Biological Psychology and Neuropsychology, University of Hamburg, Hamburg, Germany; Emory University, United States of America

## Abstract

For assessing tactile spatial resolution it has recently been recommended to use tactile acuity charts which follow the design principles of the Snellen letter charts for visual acuity and involve active touch. However, it is currently unknown whether acuity thresholds obtained with this newly developed psychophysical procedure are in accordance with established measures of tactile acuity that involve passive contact with fixed duration and control of contact force. Here we directly compared tactile acuity thresholds obtained with the acuity charts to traditional two-point and grating orientation thresholds in a group of young healthy adults. For this purpose, two types of charts, using either Braille-like dot patterns or embossed Landolt rings with different orientations, were adapted from previous studies. Measurements with the two types of charts were equivalent, but generally more reliable with the dot pattern chart. A comparison with the two-point and grating orientation task data showed that the test-retest reliability of the acuity chart measurements after one week was superior to that of the passive methods. Individual thresholds obtained with the acuity charts agreed reasonably with the grating orientation threshold, but less so with the two-point threshold that yielded relatively distinct acuity estimates compared to the other methods. This potentially considerable amount of mismatch between different measures of tactile acuity suggests that tactile spatial resolution is a complex entity that should ideally be measured with different methods in parallel. The simple test procedure and high reliability of the acuity charts makes them a promising complement and alternative to the traditional two-point and grating orientation thresholds.

## Introduction

The availability of reliable and valid psychophysical measures of tactile spatial acuity is fundamental for areas as diverse as basic research on age- and experience-dependent plasticity of spatial processing in the somatosensory system [1]–[5], clinical assessment of sensory loss and recovery of function in patients with sustained nerve damage [6] or neurological diseases [7], and rehabilitation counseling for blind people [8], amongst others (see also [9], [10]).

The two-point threshold is probably the best-known method to evaluate the spatial resolution capacity of the skin, due to its prevalent mention in textbooks and reviews on the topic (see [11], [12]). However, it has been argued that the two-point threshold might not represent a valid measure of spatial acuity because participants were assumed to discriminate one from two points on the basis of intensity cues rather than spatial cues [11], [13]. While this proposition is relevant at the level of peripheral nerve activity, recent studies focusing on cortical population activity provided evidence for a critical role of nonlinear lateral interaction processes with little evidence for differences in the magnitude of response [14], [15]. The same arguments might also apply to gap detection [13], which has been proposed as an alternative measure of tactile acuity [4], [5].

The most prominent alternative to the classical two-point threshold discussed in the literature is the grating orientation threshold (GOT). Here a grating consisting of equidistant grooves and ridges (with varying width) is presented in one of two orientations, and the participant is asked to indicate the orientation [6], [16]. The GOT has been advocated because it avoids a potential influence of response criterion and intensity cues on the measurement [9], [11], [13] and leads to relatively stable acuity estimates across measurements [16] and examiners [17]. Potential confounds in the GOT task are unknown contributions of visual cortical processing [18] and anisotropy of the task performance [19]–[21].

Despite the severe differences in the type of stimulation and the type of subject report to indicate acuity thresholds, it has been argued that the two-point threshold and GOT are roughly equivalent (see, e.g., [1]). Indeed, acuity thresholds reported at the group level have been found to be highly similar across these two methods. They usually fall between 1.2 and 1.7 mm on the fingertip for young sighted adults, and decline with age up to 3.4 mm in healthy elderly persons aged above 65 years (for an overview, see [22]). GOT [23], [24] and two-point thresholds [25] both differ between digits, with thresholds increasing from the thumb to the little finger.

All of the above-mentioned measures involve a passive presentation of brief tactile stimuli for fixed periods of time. As an alternative, tactile acuity charts (akin to the Snellen chart for visual acuity) that require active exploration of Braille patterns or raised Landolt rings of different sizes, have recently been introduced [12], [22]. A considerable advantage of these acuity charts is their easy administration, because they do not require control of contact force and duration of stimulation. This makes them particularly interesting for applied settings, such as rehabilitation counseling for the blind, or routine clinical practice, where an efficient and easy-to-use test procedure that yet allows a reliable assessment of individual treatment outcome is required [26]. Despite the advantages, the acuity charts are limited in use, as they can only be applied to the fingers and not to any other body region.

Assessment methods that involve active touch do not always result in the same conclusions as measuring tactile acuity thresholds with passive methods. For example, passive methods indicate an age-related decline of tactile acuity in blind participants as well as in sighted participants (see [22]), although thresholds are usually about 15% lower in blind participants ( [3], [8]; but see [27], [28]). Thus, standard tactile acuity measures indicate a decrease in spatial resolution with age that would bring Braille characters close to or beyond the acuity limit in the elderly. However, if tactile acuity is measured with acuity charts, tactile acuity does not seem to decline with age in blind individuals, which is in line with the observation that elderly blind people rarely lose their capability to read Braille [22]. Thus, these charts might allow for a more appropriate estimate of tactile acuity under normal perceptual conditions (see [12]). Interestingly, a similar observation was recently made using two-point threshold testing, where elderly blind participants showed almost normal acuity thresholds [29].

Overall, the apparent disagreement between methods questions the equivalence of acuity charts and passive measures, such as the two-point threshold and GOT. However, due to the lack of suitable comparison studies, it is currently unknown how active acuity measures relate to standard passive measures, and whether or not the reliability of the two procedures is comparable. The present study aimed at overcoming this limitation by directly comparing tactile acuity estimates obtained with acuity charts (as used in [22]), two-point thresholds (as used, e.g., in [1], [2]), and grating orientation thresholds (as used, e.g., in [6], [16], [30]) within the same participants. Moreover, acuity measures were obtained twice at intervals of one week, in order to derive estimates of the test-retest reliability of each measure.

## Prestudy

Since tactile acuity charts were not commercially available, we adopted the design from Legge et al. [22] and validated our stimuli in a prestudy. More specifically, we tested the robustness of the procedure to variations in the number of above-threshold characters and the properties of the material used to construct the charts. The tactile acuity charts described by Legge et al. [22] contain a relatively large number of items, including items clearly above threshold. We therefore tested in young sighted adults, whether reduced versions of these charts (testing only around threshold) would yield a quicker but similarly reliable estimate of the thresholds. This reduced set of charts was made of polymer material, which differs from standard thermo-sensitive paper (and also commercially available Braille displays) in its higher degree of softness. Electro- and photo-actuated polymer materials are promising technologies for the future development of low-cost tactile displays, including high-resolution Braille displays ( [31]; see also [32]). Therefore, we wanted to additionally assess whether this material has any adverse effects on tactile spatial resolution that might limit its usefulness in haptic displays. Indeed, it has been shown that compliant versus rigid surfaces engage distinct peripheral neural mechanisms [33]. To this end, we compared tactile acuity thresholds between the polymer charts and charts printed on thermo-sensitive paper.

### Materials and Methods

#### Ethics Statement

All participants received course credit or were paid for their participation. Written informed consent was obtained from all participants prior to the study, and the experiments were performed in accordance with the ethical standards laid down in the 2008 Declaration of Helsinki and the ethics guidelines of the German Psychological Society (DGPs). The procedure was approved by the ethics commission of the DGPs.

#### Participants

Twelve healthy sighted participants (mean age 27 years; range 20–51 years; 8 female; 9 right-handed) took part in the prestudy. Note that all except one participant were aged 30 or below.

#### Materials

**Figure 1:**
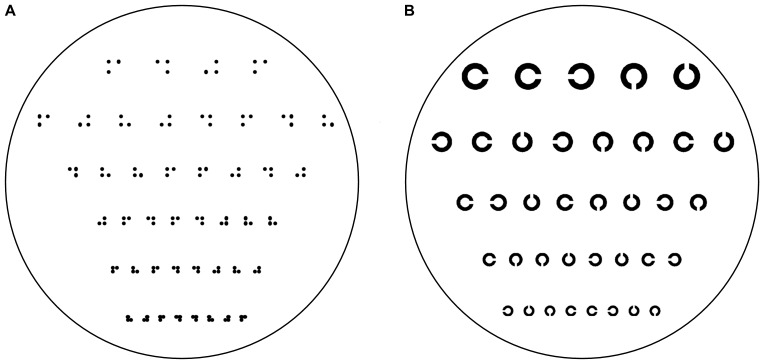
Schematic drawing of the tactile acuity charts containing dot patterns (A) and Landolt rings (B). Due to the micro-manufacturing procedure of the polymer material, the test patterns had to be fitted in a circular sheet. The size of the characters varied between lines from 0.0 (i.e., standard Braille spacing) to −0.5 log units for the dot patterns and from −0.3 to −0.7 log units for the ring patterns. The additional thermo-sensitive A4 paper versions also included lines with larger spacings up to +0.3 log units. All lines except those with the largest spacing contained 8 characters each.

Tactile acuity was measured with two types of acuity charts, containing random sequences of either three-dot patterns (corresponding to the Braille letters j, h, d, and f), or Landolt Cs in one of four orientations (see Figure 1). For each chart type, one version was printed on thermo-sensitive paper, including lines with symbols above standard Braille spacing (as used in [22]), and one version was manufactured on polymer material (see below), including only character spacing around the perceptual thresholds reported by Legge et al. [22]. The tactile acuity charts consisted of several lines of up to eight characters, which included all four character orientations with equal probability and in a randomly determined order. According to the design principles of modern logMAR visual acuity charts ( [34]; see also [22]), the character size decreased in uniform steps (i.e., on a logarithmic scale) between lines, so that the scaling factor (equal to 0.1 log units or 1.2589) was constant throughout the chart. Lines were constructed relative to a line with standard Braille spacing (2.5 mm), which was labeled 0 log units. Thus, for example, the 0.1 log unit line had a character spacing of 3.15 mm and the −0.1 log unit line had a character spacing of 1.99 mm (see also Figure 1).

All characters had an elevation of 0.4 mm. The three-dot patterns corresponded to the Braille characters j, h, d, and f and differed only in the position of the missing fourth dot (see Figure 1A). The diameter of each dot was 1 mm and remained constant on all lines of the chart, that is, only the center-to-center spacing varied between lines. Note that due to the invariant dot size, dots started to overlap for the smallest line (−0.5 log units). The Landolt rings corresponded to the Landolt C (Windows TrueType Sloan font), with the gap oriented at the top, bottom, left, or right. Note that the size of the C changes in proportion to the size of the gap (ratio 5∶1) in each row.

The polymer material used to fabricate the reduced charts was polydimethylsiloxane (PDMS; Sylgard® 184, Dow Corning, Midland, MI, USA), which is a viscoelastic material similar to rubber. The polymer charts were fabricated by a soft-molding process. First, a silicone wafer master (containing the dot and Landolt ring patterns in inverted form) was micro-machined by a deep reactive ion etching (DRIE) based process. For this purpose, an aluminum layer served as a mask, on which the dots and rings were patterned through a photolithographic process and wet etching. The PDMS was then coated onto the master, cured inside an oven, and finally removed from the master after cooling down to room temperature.

#### Procedure

The charts were placed flat on a table in front of the participants and were occluded from view by a black cloth that was supported by a frame. Participants reached beneath the cloth and read through the lines of each chart, beginning at the line with the largest characters and proceeding line by line to the smallest characters. They scanned through the lines with the index finger of their dominant hand and reported the perceived position of the missing dot or the orientation of the gap, respectively. Participants were not instructed to use a particular moving strategy and were allowed to move over the characters repeatedly. Testing was untimed, and all participants were tested on all four versions of the charts (2 character types and 2 materials), which were administered in counterbalanced order.

Tactile acuity was scored on a single-character basis according to the logarithmic scale (see [22]), that is, character size decreased by 0.1 log units (∼26%) per line, and acuity was scored in log units relative to standard Braille spacing (2.5 mm dot separation or gap width). Each missed or incorrectly recognized character was counted as 0.1 log units divided by the number of characters in that line, and the resulting sum was then added to the maximum score of −0.5 log units (dot pattern charts) or −0.7 log units (Landolt ring charts), to derive the tactile acuity score. For example, a participant who made four errors on a dot pattern chart (all in lines with eight characters) would have an acuity score of −0.5+ (4×0.0125) = −0.45 log units.

### Results

**Figure 2:**
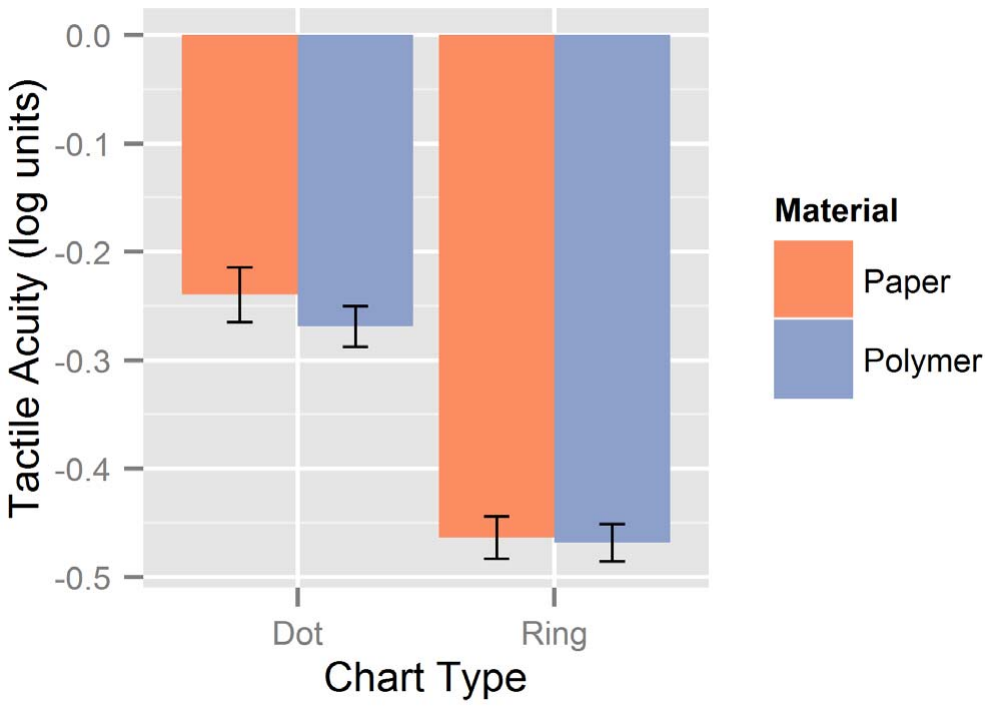
Mean tactile acuities in the prestudy. Acuity thresholds (with standard errors) obtained with the dot pattern and Landolt ring charts are shown separately for the thermo-sensitive paper (*red*) and polymer (*blue*) versions.

Overall, mean tactile acuity was higher with the Landolt ring charts than with the dot pattern charts, but did not differ between the full paper charts and the reduced polymer charts (see Figure 2). A two-way repeated-measures ANOVA with factors of Material (paper vs. polymer) and Chart Type (dot patterns vs. Landolt rings) yielded a highly significant main effect of Chart Type, *F*(1, 11) = 132.02, *p*<.001, but neither the main effect of Material nor the interaction approached significance (both *p*s>.20). Moreover, scores on the two versions of the dot pattern charts were significantly correlated, *r* = .85, *p* = .001. However, the correlation was not significant for the Landolt ring charts, *r* = .45, *p*>.10. The standard deviation of the individual differences in the acuity scores obtained with the paper and polymer charts (i.e., threshold of the paper chart minus threshold of the polymer chart) was higher for the Landolt ring charts (0.066) than for the dot pattern charts (0.048), pointing to a lower agreement between the two Landolt ring measurements (see [35]–[37]). This is consistent with the correlation analysis.

**Figure 3:**
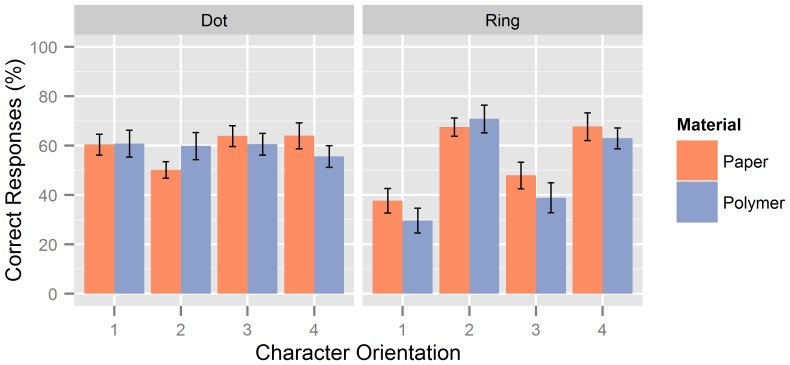
Percentage of correct identifications for the four character orientations in the prestudy. Percentages refer to correct character identifications across all lines of the polymer versions (*blue*) and across the corresponding lines of the paper versions (*red*). Character orientations refer to upper left or j (1), upper right or h (2), lower right or f (3), and lower left or d (4), for the dot charts, and top (1), right (2), bottom (3), and left (4), for the Landolt ring charts. Error bars denote standard errors of the mean.

To further assess potential differences in the reliability of the two chart types, the percentages of correct responses were analyzed separately for each of the four character orientations. Overall, hit rates were similar for the polymer and paper versions. However, with the Landolt ring charts, hit rates were higher for the gap at the left or at the right, compared to the gap at the top or at the bottom, while hit rates were similar for all four orientations of the dot patterns (see Figure 3). These observations were confirmed by separate two-way repeated-measures ANOVAs with factors of Material (paper vs. polymer) and Character (4 levels). For the dot patterns charts, neither the main effects nor the interaction were significant (all *p*s>.09). For the Landolt ring charts, neither the main effect of Material nor the interaction approached significance (both *p*s>.10), but the ANOVA yielded a highly significant main effect of Character, *F*(3, 33) = 22.22, *p*<.001. Post-hoc *t*-tests showed that hit rates for the left and right gaps were both significantly higher than for the top and bottom gaps (all *p*s<.05, Bonferroni-corrected), but hit rates did neither differ between left and right nor between top and bottom gaps (both *p*s>.10, Bonferroni-corrected).

### Discussion

The goal of the prestudy was to assess the robustness of tactile acuity measurements obtained with recently proposed acuity charts ( [22]; see also [12]) to variations in the number of above-threshold characters and to the properties of the material used. Our results demonstrate that (a) quick and reliable measurements of tactile acuity can be achieved using only a few lines around the expected acuity limit, (b) variations in surface material of the charts (polymer vs. thermo-sensitive paper) do not have an impact on acuity measurements, and (c) charts using Braille-like dot patterns seem to be more reliable than charts using Landolt rings.

Overall, mean acuity scores for the dot patterns (around −0.25 log units) and the ring patterns (around −0.45 log units) were surprisingly consistent with the values reported by Legge et al. [22] for their young sighted group and did not differ between the full paper and the reduced polymer versions. However, the suitability of acuity charts for the measurement of tactile acuity critically depends on the reliability of individual test scores as well. In this respect, the dot pattern design appeared to outperform the Landolt ring design: Individual scores on the two versions of the dot pattern chart were highly correlated and showed a relatively low variability between measurements. That is, the test-retest *SD* was in the order of three to four characters, which is comparable to the values reported in the literature on visual acuity charts (see [38]). By contrast, individual scores on the two versions of the Landolt ring chart were only weakly correlated, along with an increase of the test-retest *SD* of around 40% compared to the dot pattern charts.

The lower reliability of the Landolt ring charts might be associated with the violation of some of the well-established design principles for visual letter acuity charts, namely the use of characters with equal legibility [34] and medium difficulty [38]. Specifically, the hit rates for the Landolt rings with the gap at the top or bottom were lower than those for the Landolt rings with the gap at the left or right. It is possible that identification of the Landolt ring gaps at the top or bottom depends on the scanning strategy (e.g., the use of up-down movements in addition to lateral finger movements), which might vary across tests. By contrast, the four Braille-like dot patterns used (corresponding to the letters j, h, f, and d) showed similar hit rates around 60% correct. Moreover, according to the data reported by Loomis [39], these four characters have a medium legibility among all 26 characters of the Braille alphabet. Thus, the dot pattern charts comply very well with the design principles derived from visual acuity charts [34], [38] and appear favorable for the measurement of tactile acuity.

Finally, our results suggest that a reliable estimate of tactile acuity can be achieved by a quick screening around the expected threshold, which might help shortening the test duration in situations where reasonable presumptions about the participant’s threshold already exist. In addition, acuity measurements were not affected by the surface material of the acuity charts. This finding has obvious practical implications for the construction of tactile acuity charts, but is also of theoretical importance considering that previous studies have not systematically assessed whether tactile spatial acuity is modulated by material properties. In line with our finding, a recent study by Libouton et al. [40] obtained no significant correlation between tactile roughness discrimination performance and tactile spatial resolution, suggesting that perception of material properties and spatial resolution might be mediated by distinct neural mechanisms. Tactile spatial resolution has been associated with the SAI mechanoreceptors, whose responses and receptive fields are only marginally affected by the force of application or the indentation depth of a stimulus (see [41]). Hence, tactile spatial acuity may be largely unaffected by material properties such as softness.

## Main Study

### Materials and Methods

#### Participants

Twenty healthy sighted volunteers from the University of Hamburg participated in the main study. One participant was unavailable for the second measurement. Data of one additional participant were excluded from the analysis because the response pattern in the two-point discrimination task could not be fitted by psychometric functions, and hence no two-point threshold could be obtained. Thus, 18 participants (mean age 23.4 years; range 19–30 years; 13 female; 15 right-handed) remained in the sample. They reported normal tactile sensitivity at the fingertips.

#### Acuity charts

Due to the equivalence of measurements with the thermo-sensitive paper and polymer material versions of the charts (at least for the dot patterns), only the latter versions were used in the main study. We decided for the polymer charts because of the shorter test duration, the higher precision of the manufacturing procedure and the higher durability of the material compared to the paper versions. Tactile acuity was measured both with the dot patterns and the Landolt Cs. The procedure and scoring was the same as in the prestudy (see above). In order to compare the obtained thresholds (that are expressed in log units; see [22], [34], [38]) with the two-point threshold and GOT (that are expressed in mm), individual scores on the acuity charts were back-transformed to mm values prior to further analyses. Note that back-transformation from the logarithmic scale leads to a slight overestimation of the thresholds obtained with the acuity charts in any subsequent analysis. For example, the group average of the individual log unit scores corresponds to the geometric mean of the back-transformed data, and is thus bound to be lower than the arithmetic mean of the back-transformed data. Therefore, we chose to report the results in original log unit scores for the prestudy, as no comparison with other methods was involved in this study. However, the effect of back-transformation was negligible, and all analyses that could be performed using either the original log unit data or the back-transformed data yielded virtually identical results to those reported.

#### Two-point threshold

Spatial two-point discrimination thresholds were assessed at the index fingertip of the dominant hand using the method of constant stimuli, as described previously [1], [2], [42]–[46]. We tested seven pairs of brass needles with separations of 0.7, 1.0, 1.3, 1.6, 1.9, 2.2, and 2.5 mm. In addition, zero distance was tested with a single needle (control condition). The diameter of the needles was 0.7 mm and the diameter of the blunt endings was 200 µm. To overcome problems in the use of two-point measurements associated with hand-held probes, we used a specifically designed apparatus that secures a standardized form of testing (cf. Figure 1C in [1]). The apparatus allowed to switch rapidly between pairs of needles featuring different separations or one single needle and consisted of a disc containing the needles in front of a plate that could be moved up and down. The arm and fingers of the participants were fixed on the plate, with the test finger held in a hollow, containing a small hole through which the finger touched the needles. All tactile stimuli were applied to a fixed position on the skin of the index fingertip for approximately 1 s with an application-force of about 150 to 200 mN.

All eight test conditions were presented eight times in a randomized order resulting in 64 tests per run. The participants, who were not informed about the ratio of needle-pairs and single needles (i.e., 7∶1), had to decide immediately after removal of the stimuli whether they had the sensation of one or two needles. They were instructed to classify the percept of a single needle or doubtful stimuli as “one” but the distinct percept of two stimuli as “two”. Each stimulus was presented only once. Participants could not request repeated presentations of the stimuli before they made their judgment. The summed responses were plotted against the needle distances resulting in a psychometric function, which was fitted by a binary logistic regression (R 2.14.1; R Foundation for Statistical Computing, Vienna, Austria). The threshold was taken from the fit where 50% correct responses were reached. All participants completed three runs. For further analysis, the average of the three threshold estimates was used.

Note that false alarms (i.e., “two” responses) in the single-needle control condition (zero distance) were rare and occurred only in 5% (*SEM* = 1.6%) of the trials overall, limiting the suitability of criterion-free measures (d’) based on signal detection theory. However, two-point thresholds agreed closely (*r* = −.83) with d’ values (obtained by calculating the hit rate across all needle distances), excluding one participant who had an abnormally high false alarm rate (25%) in the control condition. Excluding this participant’s data from subsequent analyses had only marginal effects and did not change the overall result pattern. Thus, results for the complete sample (*n* = 18) are presented throughout.

#### GOT

The test procedure followed previous reports that assessed the GOT manually [6], [7], [16], [21], [23], [24], [27], [30], [47]. A set of eight hemispherical plastic domes was used (JVP Domes, Stoelting Co., Wood Dale, IL, USA), with gratings consisting of equidistant bars and grooves (0.35, 0.5, 0.75, 1.0, 1.2, 1.5, 2.0, and 3.0 mm) cut into their surface (cf. Figure 1 in [30]). The participants rested their dominant hand on a table, and the domes were manually applied to the tip of the index finger with a moderate force (i.e., with an indentation depth of about 1.0–1.5 mm) for approximately 1 s, with the grooves either parallel or orthogonal to the finger axis. Immediately following removal of the dome, participants indicated the perceived orientation verbally. Each stimulus was presented only once, and participants were instructed to guess in case they were unsure about their judgment. Note that although standardized computer-controlled application of the grating orientation task has been recommended by some authors [3], [48]–[50], manual application of the task generally leads to robust and repeatable estimates of the GOT [16], [17].

Each grating was tested in a block of 20 trials. The two grating orientations were equiprobable and their sequence within a block was randomized. Testing started with the largest grating (3 mm), and then continued with the next smaller gratings at least until performance approached chance level (50% correct) or was below threshold (75% correct) on two successive blocks. The GOT was determined by interpolating between gratings spanning 75% correct responses, unless performance was exactly 75% for a particular grating [6], [17], [21], [23], [30].

#### General procedure

Participants were tested with all three measures in two sessions at intervals of 5 to 8 days (mean 6.9 days). The order in which the acuity measures were administered was counterbalanced across participants.

### Results

#### Comparison of means

**Figure 4:**
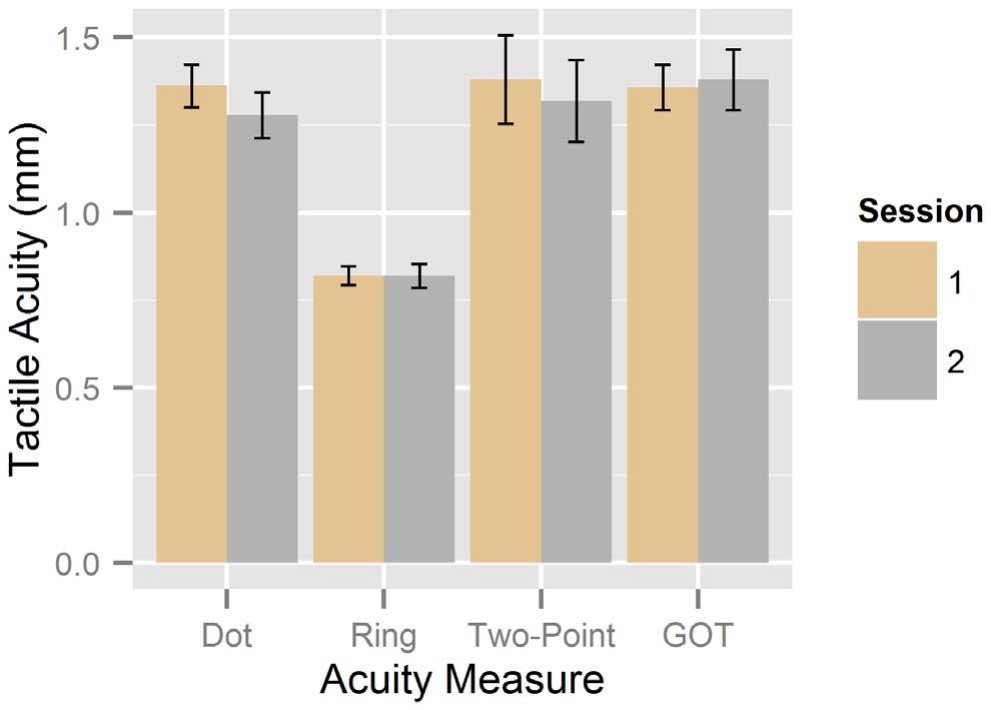
Mean tactile acuities in the main study. Acuity thresholds (with standard errors) obtained with the dot pattern and Landolt ring charts, the two-point threshold, and the GOT, are shown separately for the first (*brown*) and second (*grey*) measurement.

Mean tactile acuity thresholds obtained with the different measures are shown in Figure 4 (for individual raw data, see Table S1). Overall, mean thresholds obtained with the dot pattern chart were similar to the two-point-threshold and GOT, while the Landolt ring chart again yielded lower thresholds than the other measures. A two-way repeated-measures ANOVA with factors of Acuity Measure (dot chart, ring chart, two-point, and GOT) and Time Point (session 1 vs. session 2) suggested that measurements were not systematically influenced by repeating the tests after approximately one week, that is, neither the main effect of Time Point, *F*(1, 17) = 1.07, *p* = .315, nor the interaction, *F*<1, approached significance. A highly significant main effect of Acuity Measure was obtained, *F*(3, 51) = 16.44, *p*<.001, reflecting that thresholds were significantly lower for the ring chart than for all other measures (all *p*s<.001 after Bonferroni-correction), while there were no significant differences between the remaining three measures (all *t*s<1). Note that the mean thresholds for the acuity charts corresponded well to the results of the prestudy, that is, averages calculated on the original log unit scores were almost identical (though slightly lower) to those obtained with the polymer charts in the prestudy (dot chart: −0.29 vs. −0.27 log units; ring chart: −0.49 vs. −0.47 log units, respectively).

#### Repeatability

The test-retest reliability (i.e., the correlation between the first and second measurements), as well as the standard deviation of the individual differences between the two measurements (i.e., half the repeatability coefficient as suggested by [35]–[37]) is given in Table 1 for the different acuity measures used. The correlational analysis revealed a relatively high repeatability of the dot pattern acuity chart (.78), while all other measures had only a medium repeatability of around.6. The standard deviation of the individual differences yielded a slightly different result pattern: Repeatability was worst for the GOT, intermediate for the two-point threshold, and high for both acuity charts (with slightly higher repeatability for the Landolt ring chart). Levene’s test of equality of variances (see [51]) was significant (*p* = .004), confirming that the variability between the two measurements differed between the four test procedures. Levene’s test of equality of variances is usually used to test the assumption of homoscedasticity in classical ANOVA. However, it can also be used to test the stand-alone question whether *k* samples have equal or different variances. Here we used Levene’s test in a within-participants design (i.e., ignoring the matching) due to a lack of simple alternatives, as has been done in previous studies on the test-retest variability of visual acuity measurements [52].

**Table 1:** Repeatability Estimates for the Tactile Acuity Measures Used in the Main Study.

Parameter	Dot Chart	Ring Chart	Two-Point	GOT
*r*	.78**	.60**	.61** (.46*)	.65** (.64**)
*SD*	.18	.12	.29 (.38)	.43 (.48)

*Note. r* = correlation between acuity thresholds (in mm) obtained in the first and second session (i.e., test-retest reliability); *SD* = standard deviation of the individual differences in acuity thresholds between the first and second session (i.e., session 2 minus session 1 thresholds). For the two-point threshold and GOT, repeatability estimates based on a subset of eight trials per spacing each (matching the trial number of the acuity chart measurements) are given in parentheses.

* *p*<.05.

** *p*<.01.

The repeatability estimates might not be entirely comparable between the different methods, due to the substantial procedural differences in deriving acuity thresholds. Most notably, the acuity chart thresholds were based on a lower number of trials per spacing (8 trials) than the GOT (20 trials) and two-point thresholds (24 trials, divided in three runs with 8 trials each), which might have led to an underestimation of the relative advantage of the acuity charts over the other two methods. Therefore, we additionally calculated the repeatability for the GOT and two-point thresholds based on a subset of eight trials per spacing each, in order to match the trial number of the acuity chart measurements. In detail, the two-point threshold was derived from the first run of each session, and the GOT was derived from the first four trials for each of the two grating orientations per spacing. This analysis revealed that reducing the trial number per spacing was indeed detrimental to the repeatability of the two-point threshold: The test-retest reliability decreased from.61 to.46, along with an increase of the *SD* of the individual differences between the two sessions from 0.29 to 0.38. By contrast, reducing the trial number only marginally affected the repeatability of the GOT (see Table 1). Taken together, repeatability of the individual threshold estimates was higher with the acuity charts than with the two-point threshold and GOT procedures.

**Figure 5:**
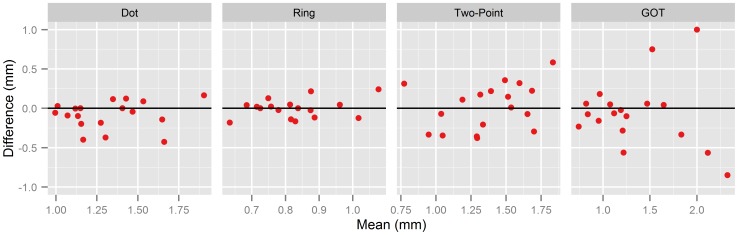
Repeated measures of tactile acuity. Panels show m-d-plots for the dot pattern and Landolt ring acuity charts, two-point thresholds, and GOT, respectively. Individual averages of the two measurements for each method are plotted against their differences (session 2 minus session 1). Horizontal lines indicate zero difference between the two measurements.

Figure 5 shows the m-d-plot (mean threshold plotted against the difference between the two measurements; see [35]–[37]) for each of the four methods. Consistent with the results of the ANOVA (see section “comparison of means” above), mean differences were not significantly different from zero for all four methods (one-sample *t*-tests, all *p*s>.05, uncorrected), confirming that tactile acuity measurements were not affected by test repetition. However, note that for the GOT differences were related to the mean of the two measurements: Repeatability decreased (i.e., differences between the two measurements were larger) for higher mean threshold values, as reflected in a significant correlation between the mean threshold and the absolute values of the differences, *r* = .70, *p = *.001. A similar relationship between the mean and differences was not observed for the other methods (all *p*s>.10).

#### Agreement between methods

Intercorrelations between the mean acuity threshold estimates (across both measurements) obtained by the four methods are presented in Table 2. Scores on the two acuity chart types were highly correlated. Both acuity charts correlated significantly with the GOT, whereas two-point thresholds were uncorrelated with all other methods. Given that empirical estimates of the test-retest reliability of each method were obtained in the present study, the correction for attenuation allows for an estimate of the intercorrelations between the methods if measurements could be obtained with perfect reliability. Technically, the correlation is divided by the square root of the product of the reliability coefficients of the two respective methods. For the correlations between the acuity charts and the GOT, this leads to an estimated “true” correlation of.66 between the dot pattern chart and the GOT, and.78 between the Landolt ring chart and the GOT. Since the two-point threshold was uncorrelated with all other measures (≤.10) the correction for attenuation had only marginal effects on the observed intercorrelations involving the two-point threshold.

**Table 2:** Summary of Intercorrelations for the Mean Acuities Obtained with the Dot Pattern and Landolt Ring Acuity Charts, the Two-Point Threshold, and the GOT.

Measure	1	2	3	4
1. Dot Chart	–			
2. Ring Chart	.90**	–		
3. Two-Point	.10	−.01	–	
4. GOT	.47*	.49*	−.02	–

*Note.* Intercorrelations were calculated on the averages across the two measurements for each method.

* *p*<.05.

** *p*<.01.

It has been pointed out in the medical literature [35]–[37] that the correlation coefficient can potentially give misleading results for the interpretation of method agreement, mainly due to the fact that the strength of the correlation depends on the variability between participants. Therefore, we additionally assessed the agreement between the four methods using the descriptive limits of agreement approach proposed by Bland and Altman [36], [37]. In this approach, agreement between two methods is quantified as the mean intra-individual difference between measurements obtained with the two methods ±1.96 *SD*s of the differences. Hence, 95% of differences between measurements by the two methods are expected to fall within these so-called limits of agreement.

**Figure 6:**
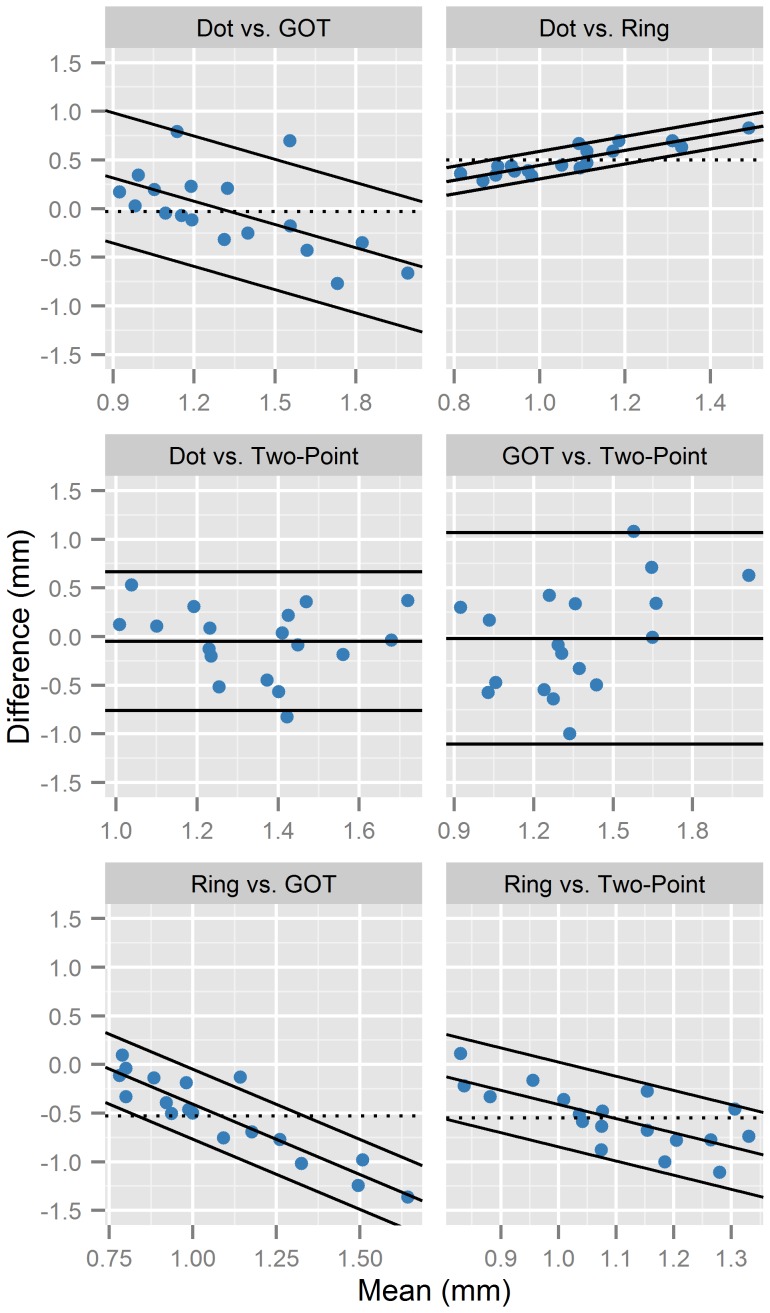
Pairwise comparisons between dot pattern and Landolt ring acuity charts, two-point thresholds, and GOT. For each of the six comparisons, m-d-plots show individual averages of the mean thresholds obtained by the two respective methods against their differences (last-mentioned minus first-mentioned method, respectively). Solid lines indicate 95% limits of agreement. For regression-based limits of agreement, dotted lines additionally indicate the mean differences between methods.

The graphical presentations of the limits of agreement (m-d-plots showing average versus difference of the mean thresholds obtained by each pair of methods) of the six pair-by-pair comparisons between the four methods are shown in Figure 6. Note that all three comparisons involving the Landolt ring acuity chart, as well as the comparison of the dot pattern acuity chart with the GOT, showed a significant linear dependence of the differences between methods on the measurement size, suggesting that the respective methods did not agree equally through the range of measurements. In these four cases, a regression-based adjustment of the limits of agreement was performed. The 95% limits of agreement are shown as the regression line ±1.96 times the residual standard deviation from the regression (see [37] for details).

Consistent with the correlational analysis, the two acuity charts showed excellent agreement, whereas the two-point threshold and GOT appeared virtually unrelated. However, for the comparisons between the two acuity charts and the traditional measures (two-point threshold and GOT), the limits of agreement approach yielded a somewhat different result pattern than the correlational analysis. For the comparisons with the GOT, the Landolt ring chart showed a reasonable agreement (as suggested by the correlational analysis). However, the limits of agreement were considerably wider for the dot pattern chart (though this difference appeared to be mainly driven by two outlier values; see Figure 6). For the comparisons with the two-point threshold, the dot pattern chart showed a poor agreement (consistent with the correlational analysis), but the agreement with the Landolt ring chart was much higher than was apparent from the correlation coefficient (though still worse than the agreement between the Landolt ring chart and the GOT). However, even for the comparison between the Landolt ring chart and the GOT, the limits of agreement analysis suggested that thresholds obtained by the two methods might differ by up to around 0.75 mm.

Finally, exploratory factor analysis was used to identify the underlying factor structure of our data, that is, whether all four methods measured the same construct of tactile acuity. According to the Kaiser-Meyer-Olkin measure of sampling adequacy, .58, and Bartlett’s test of sphericity, χ^2^(6) = 29.98, *p*<.001, factorability of the four test procedures could be assumed.

For the present study, a principal component analysis with varimax rotation was conducted, with two factors (those with Eigen values>1) explaining 82% of the variance. All four test procedures had primary loadings above.7, and the highest cross-loading was.12. The factor loading matrix for this final solution is presented in Table 3. Overall, this analysis indicated that two-point thresholds were distinct from thresholds obtained with the two acuity charts and GOT, but that the latter three (which all loaded on a single factor) represented estimates of the same underlying construct of tactile acuity. However, the relatively low communality after extraction for the GOT (.52) suggests that there was still a considerable amount of disagreement between the acuity chart thresholds and the GOT, consistent with the correlational and m-d-plot analyses reported above.

**Table 3:** Factor Loadings and Communalities Based on a Principal Component Analysis with Varimax Rotation for the Four Tactile Acuity Measures.

Measure	1	2	Communality
Dot Chart	**.93**	.12	.88
Ring Chart	**.94**	.00	.89
Two-Point	.01	**.99**	.99
GOT	**.71**	−.08	.52

*Note.* Factor loadings>.70 are in boldface.

### Discussion

The main study directly compared tactile acuity estimates obtained with the acuity charts with the more established two-point and grating orientation thresholds within the same participants. Moreover, the test-retest reliability of each measure was assessed. Our results showed that the repeatability of tactile acuity thresholds (measured at intervals of one week) obtained with the dot pattern and Landolt ring acuity charts was overall better than the repeatability of the two-point threshold and GOT. Both acuity charts agreed reasonably with the GOT, but only weakly with the two-point threshold, which was also relatively unrelated to the GOT. The implications of these findings are considered in the General Discussion.

Measurements with the two chart types were highly equivalent, although thresholds obtained with the Landolt ring chart were generally lower than thresholds obtained with the dot pattern chart, consistent with the results of our prestudy and the results reported by Legge et al. [22]. This difference between the two chart types has been attributed to the design of the characters: Dot size remains constant and thus dots start to overlap for small spacings, whereas the Landolt ring stimuli scale in size, which might contribute to increased legibility for small spacings; in addition, spacing is defined as center-to-center for the dot patterns, but as edge-to-edge for the Landolt ring gaps, which tends to penalize performance on the dot chart [22]. Interestingly, however, differences between measurements with the Landolt ring chart and the other methods seemed to be stronger for participants with higher threshold values (see Figure 6), which is somewhat counterintuitive if differences between the two chart types are attributed to the elimination of a floor effect on the ring charts, as suggested by Legge et al. [22]. Nevertheless, given that measurements with the two chart types agreed well, the mean acuity differences seem to be of minor importance for the suitability of the charts to measuring individual differences in tactile acuity.

Contrary to the results of the prestudy, a clear difference in the repeatability of the two chart types was only found for the correlation coefficient, but not for the standard deviation of the differences between the two sessions [35]–[37]. However, as in the prestudy, differences in the hit rates for the four character orientations were observed for the Landolt rings, but not for the dot patterns (see Figure S1), supporting the conclusion that the design of the Landolt ring chart might lead to less reliable estimates of tactile acuity than the design of the dot pattern chart.

## General Discussion

Recently, tactile acuity charts (akin to the Snellen letter charts for visual acuity) have been suggested as an alternative measure of tactile spatial resolution [12], [22]. Unlike traditional measures that require controlled passive stimulation, these charts involve active exploration of simple dot patterns or Landolt rings with different orientations, thereby largely eliminating potential influences due to the experimenter as well as the need for special apparatuses that ensure controlled stimulation. On the one hand, our results show that this simple and straightforward procedure yields highly repeatable estimates of tactile acuity that agree reasonably with the GOT, but less so with the two-point threshold. On the other hand, our results indicate a potentially considerable amount of disagreement between different measures of tactile acuity, suggesting that studies on tactile spatial resolution should ideally measure tactile acuity with different methods in parallel.

Primarily, tactile spatial resolution is mediated by the innervation density of the slowly adapting Merkel (SAI) afferents [41]. Accordingly, SAI receptive field spacing seems to be the main determinant of both the GOT [6], [16], [53], [54] and two-point threshold [55]–[57]. Other peripheral factors such as skin conformance have rather marginal effects on tactile acuity ([53], [58]; though see [21]). Although innervation density of SAI afferents incontrovertibly accounts for a large amount of the inter-subject variability [53], tactile spatial acuity depends on central processing of the peripheral neural image as well.

Using both functional magnetic resonance imaging (fMRI; [44]) and source reconstruction of somatosensory-evoked brain potentials [59], [60], it has been shown that perceptual improvements in tactile acuity due to tactile coactivation (a simple form of Hebbian learning involving repetitive and synchronous stimulation of the receptors on the fingertip) are associated with an enlargement of the index finger representation in the primary somatosensory cortex (SI). This result points towards a crucial role of cortical reorganization for changes in tactile acuity. Moreover, transcranial magnetic [46] and direct current stimulation [61] over SI led to similar enhancements of tactile acuity as induced by tactile coactivation. Likewise, the decline in tactile acuity typically observed in elderly people has been associated with cortical map alterations [43] rather than peripheral changes [21]. Finally, the observation that mere visibility of the stimulated limb can enhance tactile acuity [62] via a modulation of activity in SI [63], suggests that cortical maps might be dynamically adapted even at very short timescales.

Use-dependent improvements of tactile acuity, as have been observed behaviorally in blind individuals [3], [8], [22], [30], [49], professional pianists [45], and even Tai Chi practitioners [47], are likely to depend on such central reorganization processes as well. However, results on use-dependent plasticity have been mixed. For example, enhanced tactile acuity in blind compared to sighted individuals has not been reported consistently [27], [28]. Moreover, manual dexterity expertise, as required in opticians, goldsmiths, dentists, watch makers, or hearing care professionals, seems to be unrelated to tactile acuity thresholds [64]. Considering that most studies assessed use-dependent plasticity of tactile acuity using a single behavioral outcome measure (either the two-point threshold or the GOT), the question arises to what extent conflicting results might be due to suboptimal repeatability or validity of the psychophysical test procedures used, as implied by the results of our study. As a striking example, two recent studies (using standard manual application of the GOT as in our study) reported that short-term light deprivation in sighted individuals leads to similar enhancements of tactile acuity as have been observed in blind individuals [65], [66]. However, Wong et al. [50] failed to replicate this finding using computer-controlled application of the GOT [48] in a much larger sample size, and attributed this inconsistency (at least partially) to the presumably higher reliability of automated assessments of the GOT compared to manual assessments.

The manual test procedure for the GOT has been used widely [6], [7], [16], [21], [23], [27], [28], [30], [47], [64]–[66], presumably due to the commercial availability of the stimulus material. However, based on our findings showing only medium repeatability of the GOT with manual application and partial agreement with other measures of tactile acuity, and the findings of Wong et al. [50], it seems advisable to include additional measures of tactile acuity in any future study on this topic. The tactile acuity charts, due to their quick and easy administration and reasonable agreement with the GOT in the present study, would be a suitable candidate for validating results obtained with the GOT. Moreover, it is conceivable that automated or semi-automated assessment of the GOT [3], [48]–[50],[53],[58],[61] improves the reliability of the measurement and should be preferred whenever possible.

The two-point threshold, unlike the GOT, showed only poor agreement with the tactile acuity charts, and, in addition, was unrelated to the GOT in the present study. On first glance, this finding seems to confirm earlier criticisms of the two-point threshold as an invalid measure of tactile spatial acuity [11], [13]. However, it should be considered that in the present study two-point thresholds were based on only three measurements per participant and session, whereas most studies that used the two-point threshold as a behavioral indicator of tactile acuity obtained thresholds from many repeated measurements [1], [2], [42]–[46], [59], [60], [67]. Under such circumstances, a much higher test-retest reliability of around.9 has been observed for the two-point threshold [1], although measured at shorter intervals than in the present study (days rather than one week). This is consistent with our observation that the test-retest reliability decreased considerably if the two-point threshold was derived only from the first measurement, rather than from the average of all three measurements. Moreover, if both two-point thresholds and GOT are based on repeated measurements (rather than a single measurement of the GOT as in the present study), a reasonable correlation of around.7 has been observed between the two measures (unpublished data, see, e.g., [1]). This might suggest that different tactile acuity measures converge to a larger degree with higher numbers of stimulus presentations (that would presumably reduce noise in the measurements). Particularly the repeatability of the two-point threshold seems to benefit from increasing the trial number, whereas the repeatability of the GOT was only marginally affected by the number of stimulus presentations in our study.

Given that repeated measurements, as well as automated stimulus presentation, could potentially enhance repeatability of the two-point threshold and GOT, and considering the relatively small sample size of the present study, our results should be interpreted as lower boundaries for repeatability and method agreement of tactile acuity measures. Nevertheless, at least parts of the observed intra-subject variability between methods are likely due to assumed massive differences in the underlying neural responses elicited by substantially different forms of stimuli. In visual cortex, presentation of a small point-like stimulus evokes a small focused activation. When instead two small point-like stimuli were presented at various separations, mimicking the conditions during tactile two-point stimulation, evidence for distance-dependent nonlinear lateral interaction processes was found [15]. Similar observations were obtained in somatosensory cortex for tactile stimulation [14], arguing against a simple intensity-based mechanism underlying the behavioral two-point threshold [11], [13]. By contrast, little is known about somatosensory cortical representations of oriented gratings [68]. Visual presentation of an oriented grating evokes a highly complicated 2-dimensional pattern of cortical activation. Perceptually, spatial resolution measured with oriented gratings depended on the orientation, and this effect varied across the visual field [69]. Similar anisotropies have been reported for tactile gratings [19]–[21], indicating that a simple relation between acuity and orientation might underestimate the complexity of cortical processing.

Unlike both two-point and grating orientation discrimination, the tactile acuity charts allow active scanning of Braille-like dot patterns or Landolt rings. Active scanning recruits rapidly adapting (RA) afferents, which allows the transmission of spatial information in the form of a temporal modulation of RA responses in addition to the spatial code conveyed by the SAI afferents [70]. Such a temporal code seems to underlie the representation of fine textures in the peripheral nerve, but might, however, not be sufficient for dissolving their spatial layout [71] as required in the tactile acuity charts. It has been argued that the RA signal might even have adverse effects in spatial acuity tasks due to an interference with the spatially modulated signal conveyed by the SAI afferents [72]. Thus, the innervation density of the SAI afferents might be the limiting factor for spatial acuity during both active and static touch. Accordingly, it has been shown that the impulse rate of SAI afferents increases with no loss of spatial resolution during active versus static touch [73], [74].

Using fMRI, greater activation was also found in contralateral SI during active touch as compared to passive touch in a tactile roughness categorization task [75]. Apart from differences in terms of activation strength, animal studies have shown that ensemble activity recorded in each layer of the whisker area of SI while rats performed a whisker-dependent tactile discrimination task is fundamentally different from activity evoked by similar passive whisker stimulation. Moreover, significant layer-specific functional differences in SI activity were observed during active discrimination. These differences appeared unlikely to be due to variations in ascending thalamocortical inputs. Instead, results suggested a top-down modulation during active discrimination [76]. Similarly, the use of active versus passive touch was associated with a refinement of neural representations in the somatosensory cortex during tactile discrimination learning [77]. Possibly, active measures of tactile acuity are more sensitive to such use-dependent central changes than traditional passive measures.

Taken together, our results show that tactile acuity charts yield highly repeatable estimates of tactile acuity that correspond reasonably to the GOT, which might be considered the current gold standard. While the dot pattern and Landolt ring versions had a very high agreement in the main study, we would recommend the use of the dot pattern chart due to its overall better repeatability and conformance with the design principles derived from visual acuity charts. Due to the quick and relatively easy and fail-safe application of the acuity charts, they provide a promising alternative to traditional passive measures of tactile spatial acuity.

## Supporting Information

Figure S1 Percentage of correct character identifications for the acuity charts in the main study. Percentages refer to correct character identifications across all lines of the charts, averaged across the two measurements. Character orientations refer to upper left or j (1), upper right or h (2), lower right or f (3), and lower left or d (4), for the dot chart, and top (1), right (2), bottom (3), and left (4), for the Landolt ring chart. Error bars denote standard errors of the mean. With the Landolt ring chart, hit rates were higher for the gap at the left or at the right, compared to the gap at the top or at the bottom (all *p*s<.05, Bonferroni-corrected), but hit rates did neither differ between left and right nor between top and bottom gaps (both *p*s>.05, Bonferroni-corrected). Hit rates were similar for all four orientations of the dot patterns (all *p*s>.05, Bonferroni-corrected).
(PDF)

Table S1 Individual tactile acuity thresholds (in mm) obtained with the dot pattern and Landolt ring acuity charts, the two-point threshold, and the GOT.
(PDF)
