# Supplementary figures and images for: Tactile Acuity Charts: A Reliable Measure of Spatial Acuity
Source: PLoS One. 2014 Feb 4;9(2):e87384. doi: 10.1371/journal.pone.0087384 (PMC3913609; doi:10.1371/journal.pone.0087384)

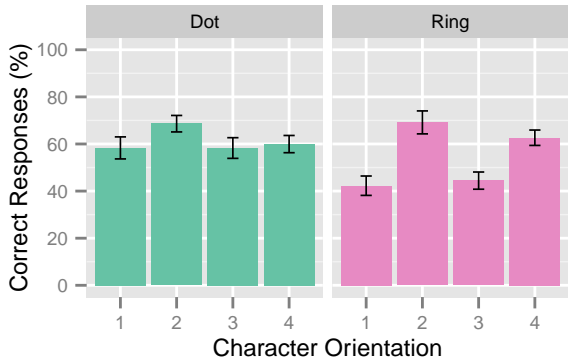

Supplement: Figure S1 — Percentage of correct character identifications for the acuity charts in the main study. Percentages refer to correct character identifications across all lines of the charts, averaged across the two measurements. Character orientations refer to upper left or j (1), upper right or h (2), lower right or f (3), and lower left or d (4), for the dot chart, and top (1), right (2), bottom (3), and left (4), for the Landolt ring chart. Error bars denote standard errors of the mean. With the Landolt ring chart, hit rates were higher for the gap at the left or at the right, compared to the gap at the top or at the bottom (all ps<.05, Bonferroni-corrected), but hit rates did neither differ between left and right nor between top and bottom gaps (both ps>.05, Bonferroni-corrected). Hit rates were similar for all four orientations of the dot patterns (all ps>.05, Bonferroni-corrected). (PDF) [file pone.0087384.s001.pdf]
