# Supplementary material for: Tactile Acuity Charts: A Reliable Measure of Spatial Acuity
Source: PLoS One. 2014 Feb 4;9(2):e87384. doi: 10.1371/journal.pone.0087384 (PMC3913609; doi:10.1371/journal.pone.0087384)
Supplement: Table S1 — Individual tactile acuity thresholds (in mm) obtained with the dot pattern and Landolt ring acuity charts, the two-point threshold, and the GOT. (PDF) [file pone.0087384.s002.pdf]

Table S1.

Individual tactile acuity thresholds (in mm) obtained with the dot pattern and Landolt ring acuity charts, the two-point threshold, and the GOT.

	Dot Chart		Ring Chart		Two-Point		GOT	
Participant	S1	S2	S1	S2	S1	S2	S1	S2
1	1.49	1.12	0.95	0.83	0.62	0.93	1.05	1.10
2	1.00	1.02	0.73	0.73	1.54	2.13	0.88	0.80
3	1.49	1.58	0.84	0.84	1.23	1.40	0.86	0.63
4	1.02	0.97	0.73	0.54	1.44	1.59	0.88	1.06
5	1.82	1.99	0.96	1.20	1.53	1.54	1.35	1.07
6	1.25	1.05	0.70	0.73	1.22	0.87	1.44	1.50
7	1.37	1.49	0.75	0.77	1.28	1.50	1.50	0.94
8	1.18	1.09	0.68	0.81	1.44	1.23	1.30	1.20
9	1.12	1.02	0.73	0.73	1.12	0.78	1.15	1.09
10	1.12	1.12	0.67	0.70	1.57	1.80	1.20	1.18
11	1.29	1.41	0.89	0.86	1.07	1.00	2.40	1.83
12	1.15	1.15	0.79	0.84	1.44	1.76	1.03	0.88
13	1.37	1.18	0.91	0.75	1.13	1.24	1.15	1.90
14	1.49	1.45	0.77	0.98	1.69	1.62	1.63	1.67
15	1.37	0.97	0.79	0.77	1.48	1.10	0.79	0.85
16	1.72	1.58	1.08	0.96	1.47	1.11	1.50	2.50
17	1.41	1.41	0.89	0.75	1.31	1.67	2.00	1.67
18	1.87	1.45	0.94	0.98	1.85	1.55	2.75	1.90

Note. S1 = session 1; S2 = session 2.
